# Exploring a New Simulation Approach to Improve Clinical Reasoning Teaching and Assessment: Randomized Trial Protocol

**DOI:** 10.2196/resprot.4938

**Published:** 2016-02-17

**Authors:** Thomas Pennaforte, Ahmed Moussa, Nathalie Loye, Bernard Charlin, Marie-Claude Audétat

**Affiliations:** ^1^Sainte-Justine HospitalDepartment of NeonatologyUniversity of MontrealMontreal, QCCanada; ^2^Faculty of EducationDepartment of Administration and Foundations of EducationUniversity of MontrealMontreal, QCCanada; ^3^Centre for Applied Pedagogy in Health SciencesUniversity of MontrealMontreal, QCCanada; ^4^Faculty of medicine(UIGP) Primary Care UnitUniversity of GenevaGenevaSwitzerland

**Keywords:** clinical reasoning, simulation, debriefing, iterative discussions, diagnostic errors, cognitive bias, verbalization, dual-process theory

## Abstract

**Background:**

Helping trainees develop appropriate clinical reasoning abilities is a challenging goal in an environment where clinical situations are marked by high levels of complexity and unpredictability. The benefit of simulation-based education to assess clinical reasoning skills has rarely been reported. More specifically, it is unclear if clinical reasoning is better acquired if the instructor's input occurs entirely after or is integrated during the scenario. Based on educational principles of the dual-process theory of clinical reasoning, a new simulation approach called simulation with iterative discussions (SID) is introduced. The instructor interrupts the flow of the scenario at three key moments of the reasoning process (data gathering, integration, and confirmation). After each stop, the scenario is continued where it was interrupted. Finally, a brief general debriefing ends the session. System-1 process of clinical reasoning is assessed by verbalization during management of the case, and System-2 during the iterative discussions without providing feedback.

**Objective:**

The aim of this study is to evaluate the effectiveness of Simulation with Iterative Discussions versus the classical approach of simulation in developing reasoning skills of General Pediatrics and Neonatal-Perinatal Medicine residents.

**Methods:**

This will be a prospective exploratory, randomized study conducted at Sainte-Justine hospital in Montreal, Qc, between January and March 2016. All post-graduate year (PGY) 1 to 6 residents will be invited to complete one SID or classical simulation 30 minutes audio video-recorded complex high-fidelity simulations covering a similar neonatology topic. Pre- and post-simulation questionnaires will be completed and a semistructured interview will be conducted after each simulation. Data analyses will use SPSS and NVivo softwares.

**Results:**

This study is in its preliminary stages and the results are expected to be made available by April, 2016.

**Conclusions:**

This will be the first study to explore a new simulation approach designed to enhance clinical reasoning. By assessing more closely reasoning processes throughout a simulation session, we believe that Simulation with Iterative Discussions will be an interesting and more effective approach for students. The findings of the study will benefit medical educators, education programs, and medical students.

## Introduction

### Background

#### The Importance of Clinical Reasoning in Medicine

Diagnostic errors account for more than 8% of adverse events in medicine and up to 30% of malpractice claims [1]. These errors may be related to the working environment but clinical reasoning issues are involved in about 75% of the cases, either alone or in association with system failures [2].

In this context, clinical reasoning is a crucial skill for all physicians regardless of their area of expertise and becomes a central aim of medical education [3]. The clinical reasoning process is largely supported by several decades of research in cognitive psychology and has been extensively published [4-6]. The most accepted model of clinical reasoning is called *dual-process framework* and consists of two independent systems [7]. System-1 is automatic, intuitive, nonanalytical, and error-prone. It leads to the immediate recognition of the clinical constellation and the generation of a working diagnostic hypothesis. System-2 is slower, more analytical, and conscious [8,9]. Novices employ this analytic mode of reasoning more frequently than their experienced counterparts because they lack the necessary experience for System-1 reasoning. Dual-process theory posits that both systems are simultaneously required in most clinical scenarios and has been associated with better diagnostic outcomes [10]. However, in what situations does the valence go towards one system or another remains unclear and how both systems are activated and used is still under study and debated [11-13]. Preliminary conclusions from recent publications describe that the analytical system is primarily used in the following situations [14,15]: when time permits, when there are high-stakes outcomes, when the situation is complex, when the decision-maker is facing ambiguous, nonroutine or ill-defined problems, and in the context of uncertainty. In contrast, routine problems associated with a higher level of certainty would be more often dealt with by the intuitive system, especially when time is lacking [11-13].

Another challenge for medical educators is the assessment of residents’ clinical reasoning [3]. Because of the paucity of scientific evidence about optimal evaluation, both quantitative and qualitative clinical reasoning assessment tools have been reported [5,16-19]. However, the following general issues arise from these tools: (1) diagnostic reasoning must be inferred from behavior because it is not a discrete and measurable quality; (2) most of these instruments are performed in the classroom that emphasize the assessment of System-2, but not System-1 reasoning nor the shift between automatic and analytic reasoning [15,16]; and (3) rater’s personal knowledge, experience, ability, and cognitive biases influence his or her adjudication of a learner’s performance in a nonstandard fashion [20-24]. Moreover, reasoning assessment can be highly complex and dependent upon the context. Durning et al [25] have recently reported the influence of three environmental factors on clinical reasoning: (1) the patient’s specific problem (patient factors); (2) the setting in which the patient is evaluated (encounter factors) [10,26]; and (3) human-factors such as fatigue, well-being, and sleepiness (doctor factors). They emphasize the importance of measuring the environment as a part of the signal, rather than part of the “noise”, which is to be minimized and generally ignored [27-29].

#### Simulation-Based Education as a Strategy to Enhance Clinical Reasoning Skill

Simulation-based education (SBE) has recently emerged as an instrument with potential to assess diagnostic reasoning [16,30,31]. Based on Kolb’s learning cycle [32], true learning is depicted as a four-part process in a cycle. Individuals learn through concrete experience (phase 1), reflection on the experience (phase 2), conceptualization of their reflective observations into more abstract models (phase 3), and experimentation of these new principles and conclusions to guide subsequent decisions and actions that lead to new concrete experiences (phase 4). Phase 2 and phase 3 are components of debriefing, a learning activity that generally follows a simulation experience. According to many authors, debriefing could provide an opportunity for residents and faculty to re-examine what occurred during the simulation process and detect possible flows in the reasoning process [33-45].

An important question remains whether exploration of the diagnostic process, by providing an opportunity for learners to reflect upon past clinical decisions, is more effective after the scenario or if it is integrated during the simulation session [45,46]. In the classical approach of simulation, debriefing follows the simulation experience. However, the educational valence of this type of session presents several limitations concerning the clinical reasoning assessment. First, this way of *reflection-on-action* means that it is mainly the analytical part of the reasoning process that is explored during the debriefing, without focusing on the intuitive process [47]. Second, after a stressful scenario, residents frequently forget or modify what they said or thought according to the evolution of the case, even if video recordings provide insight into what may not be documented in the medical record or fully observed in real time [45].

We believe that changes in the organization of a simulation session could allow better assessment of both System-1 and 2 of the reasoning process. First, *reflection-in-action* should be encouraged by concurrent verbalization to let the tutors know about the student’s intuitive System-1 thinking during management of a patient [11-13]. Second, *reflection-on-action,* which reflects analytical System-2 should be encouraged by in-simulation interruptions at key moments of the clinical reasoning process [48,49]. These interruptions could be assimilated to a dynamic and decision-dense environment where clinical reasoning constructs must be considered, as studies suggest that the average time on particular tasks is limited to less than 2 minutes, and interruptions occur every 2 to 10 minutes in the emergency department [50-52]. Finally, the active experimentation of newly acquired conceptualizations in a subsequent part of the scenario may avoid the learner’s return to actions based on habits and nonreflective experience [32]. Based on these arguments, we present a new approach of SBE, called simulation with iterative discussions (SID). The simulation session is designed as a single scenario with a computerized mannequin where the instructor interrupts the flow of the scenario at three key moments to cue residents towards the appropriate medical management of the case. The objectives of the session are to build a scaffold of clinical reasoning competencies throughout the scenario while continuing to manage the patient, and to improve concept acquisition and retention with spaced learning. We believe that a closer assessment of both System-1 (by concurrent verbalization during patient management) and System-2 (during iterative discussions) will improve students’ capability to self-improve clinical reasoning skills in an authentic setting.

#### Why Is It Applied in Neonatology?

Implementing a new educational strategy that assesses clinical reasoning should be particularly exciting in a busy clinical environment such as neonatology. In contrast to the well-established management of neonatal emergencies at birth [53], most daily clinical situations managed in neonatology are nonroutine or ill-defined problems marked by high levels of complexity and unpredictability, requiring that clinical reasoning is solely based on the analytical System-2 [3,6,14,15,54]. Moreover, the frequency and the impact of diagnostic errors and cognitive biases increase in emergency settings due to several factors such as stress, fatigue, circadian disruptions, time constraint, and noisy environment. In these high-risk situations, physicians rely on System-1 process [55-58]. By enabling both System-1 and 2 of clinical reasoning, practicing neonatology requires robust clinical reasoning abilities in addition to cognitive, technical, and behavioral skills.

### Aim of the Study and Working Hypotheses

The aim of this study is to explore how clinical reasoning abilities of residents in General Pediatrics and Neonatal-Perinatal Medicine evolve and are learned with the SID in comparison to the classical approach of simulation.

We hypothesize that (1) SID allows better assessment of both System-1 and 2 of clinical reasoning; (2) SID promotes higher self-progression of the clinical reasoning process when compared to the classical approach of simulation; (3) concurrent verbalization benefits mainly novice residents with underdeveloped System-1 reasoning process; and (4) iterative discussions benefit both novice and expert residents by enhancing System-2 processes.

## Methods

### Setting and Population

The study will take place at the Mother-Child Simulation Center at CHU Sainte-Justine, between January and March 2016. CHU Sainte-Justine is a standalone pediatric center that houses a 65 bed level 3 Neonatal Intensive Care Unit (NICU). The simulated setting will be a NICU. The simulation center is equipped with appropriate audio-visual equipment.

Residents enrolled in the General Pediatrics and Neonatal-Perinatal Medicine programs will be the target population for this study. This population is selected because (1) residents are regularly exposed to simulation training during their curriculum, and (2) their clinical reasoning ability improves between the 1^st^ and the 6^th^ year of residency, mainly with increasing clinical exposure through rotations [59]. All postgraduate year (PGY) 1 to 6 residents enrolled in the General Paediatrics and Neonatal-Perinatal Medicine programs at Université de Montréal between May 1 and June 30, 2015 will be eligible for inclusion. There will be no exclusion criteria.

### Ethical Considerations

Each resident will be approached for consent by one of the authors. He/she will be informed that participation or lack of participation in the study will not impact residency training assessment. There will be no financial incentive to participate, and participants will be able to opt out at any moment of the study. The findings of this study will be treated anonymously. The project has been approved by CHU Sainte-Justine’s institutional review board on July 15, 2014.

### Study Design

This is a prospective exploratory nonblinded randomized mixed-methods study. Both quantitative and qualitative research methods will be used simultaneously as to comprehensively explore how clinical reasoning develops through both simulation modalities.

**Figure 1:**
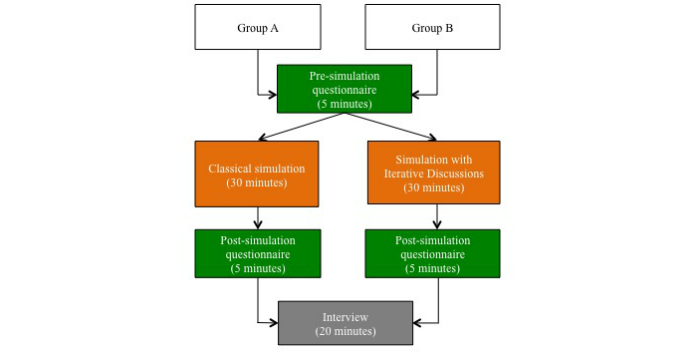
Study protocol.

Randomization will be stratified according to two different groups depending on their level of exposure to the NICU: (1) novice residents (PGY1 and 2, exposed to less than 8 weeks to the NICU), and (2) expert residents (PGY3-6, exposed to at least 8 weeks to the NICU). These residents have been respectively exposed to at least 5 or 15 simulation sessions during their residency training. Residents will be randomly (by draw of names) allocated by the primary investigator to group A or group B (Figure 1). Group A will be exposed to the SID approach, whereas group B will be exposed to the classical approach of simulation. Participants will be scheduled to complete the study protocol in 60 minutes. In the first 5 minutes, participants will complete the presimulation questionnaire and receive a brief introduction including a period to get physically familiarized with the mannequin (SimNewB; Stavanger, Norway). In the next 30 minutes, residents will complete the clinical simulation scenario after reading a clinical vignette. At the end of the session, participants will have 5 minutes to complete the postsimulation questionnaire (see Multimedia Appendix 1). The course will end with a 20 minutes semistructured interview.

### Intervention

#### Personnel

An instructor experienced with programming, control of the computerized mannequin, and the art of debriefing will be in charge of running the scenario according to the residents’ actions and will conduct the iterative discussions and debriefings. A facilitator will also be present in the simulation room to ensure the flow of the scenario and will provide necessary information about the case upon request from the resident. Standardized health professionals (respiratory therapist and/or a nurse working in the Sainte-Justine hospital NICU) will be present and will portray a specialist from their proper field.

#### Description of the Intervention

**Figure 2:**
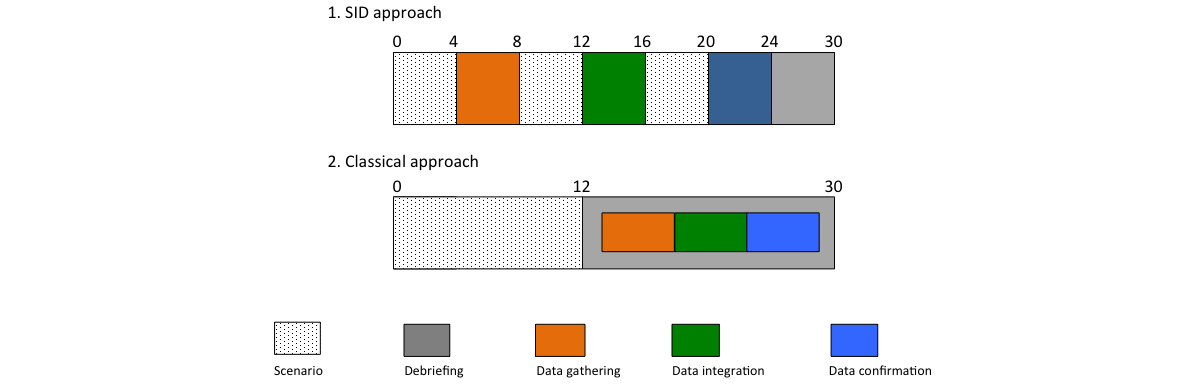
Simulation formats. This figure represents structures of (a) SID and (b) classical approach of simulation, with approximate timing in minutes. (a) A three-time interrupted scenario, with three stops that represent iterative discussions concerning data gathering, data integration, and data confirmation. There should be no guidance by the instructor until the real and short debriefing ending the session. (b) A one-shot scenario followed by a true debriefing conducted by the instructor according to the item checked in the clinical reasoning assessment tool. As a standard debriefing, retroaction from the instructor will be possible.

The SID approach (Figure 2a) consists of a scenario interrupted at three moments and followed by a short debriefing. According to Kuhn’s steps of the medical reasoning process [60], the session is divided into “data gathering”, “data integration,” and “data confirmation”. Each part consists of two phases. First, the participant is asked to manage a simulated patient based on a real-life and complex case. Complexity, uncertainty and environmental factors such as doctor, patients, and encounter factors [25] are voluntarily embedded. The participant is also asked to verbalize his or her first *intuitive* diagnosis as it comes in mind by System-1 activation. Second, the scenario stops and the instructor questions the participant on his clinical reasoning process at that point in time by System-2 activation. Each interruption must be as short as possible in order to keep the trainee in action, and is ended by a one-sentence reconceptualization of the scenario by the instructor before pursuing the scenario (for the two first stops). Discussions include questions regarding data gathering and the rationale of ordered investigations (first stop), data integration, and how investigation results helped reach a diagnosis (second stop), and finally data confirmation and how the management decisions were reached (third stop). There is neither feedback nor guidance from the instructor during the stops in order to not interfere with the participant’s ongoing clinical reasoning process. These stops are “discussions” and not “debriefings”. A short general debriefing ends the session, and provides feedback on reasoning, procedural skills, and knowledge by highlighting the learnt key messages.

The classical approach (Figure 2b) consists of a scenario with no intervention provided by the instructor until debriefing. As in the SID approach, the participant must verbalize his reasoning process and the diagnostic hypotheses as they come to mind during the scenario. Immediately after the scenario, a facilitated debriefing is performed. This debriefing lasts two to three times the length of the scenario and focuses on the participant’s clinical reasoning skills.

#### Clinical Reasoning Assessment Tool

**Figure 3:**
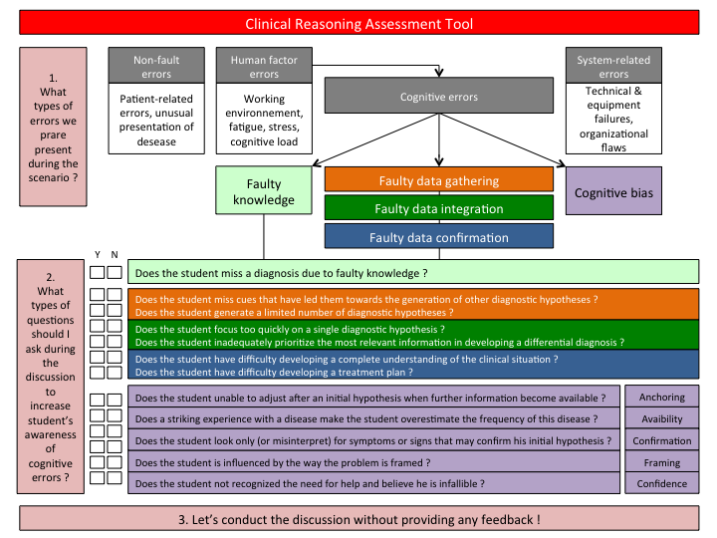
Clinical reasoning assessment tool is a useful tool for detecting diagnostic errors (such as nonfault, human factors, cognitive, and system-related) and clinical reasoning difficulties according to Kuhn classification (data gathering, data integration, and data confirmation). Instructor has to read questions for each category of error or difficulty and compare with the student performance during the simulation session. By checking all sort or errors concerning clinical reasoning, this tool permit to build specific questions for the student (without feedback for SID approach) or to construct his debriefing (with feedback for classical approach of simulation).

The Clinical Reasoning Assessment Tool (Figure 3) aids the instructor to identify proper questions during the iterative discussions (SID approach) and in conducting debriefing (classical approach). The authors of the present study have developed this tool because of the absence of such a tool in the literature. It is based on Graber’s classification of diagnostic errors [2,25], Audétat’s practical guide [61] to assist clinical teachers in detecting clinical reasoning difficulties and follows Kuhn’s steps of the medical reasoning process [60]. The objective of this tool is to help the instructor detect the student’s type of diagnostic errors focusing on his reasoning process, and then to determine appropriate questions to ask the student in order to let him verbalize and possibly self-correct his reasoning process. Three types of environmental factors leading to diagnostic errors may be involved in the reasoning process: nonfault factors (also named patients errors, which are out of the control of the physician), human factors and cognitive factors (also named doctor errors) and system factors (also named encounters errors, due to organizational or institutional flaw). Specific failure in the doctor’s cognitive process may be due to faulty knowledge, faulty recognition of cognitive biases or faulty data gathering (orange squares), data integration (green squares), and data confirmation (blue squares). One or two questions per section are suggested to the instructor to allow for exploration of each type of diagnostic error. Finally, a list of the main cognitive biases according to Croskerry et al [62] is provided and a suggested approach is presented. Once the type of error is identified, the instructor has to find one or two specific questions in order to let the student verbalize (or self-correct) his reasoning mistake.

#### Scenario

In order to stimulate the reasoning process, the chosen topic has to be realistic, and hold a range of differential diagnoses. Investigations should be necessary to precise or refute diagnoses in the absence of specific clinical signs. Uncertainty needs to be deliberately embedded. Management must be complex with controversies concerning treatments. Moreover, a range of environmental factors and events can be added to challenge team members by generating dissonance and failures in order to optimize efficiency of simulated team training and adult learning. The amount of provided information has to be minimal and nonspecific aiming to stimulate additional questions from the participant. Answers to participants’ questions must be standardized and should cover a range of possible differential diagnoses. Finally, procedural and relational skills must be embedded in order to portray as closely as possible the real-life environment.

The general structure of the scenario must follow three parts so it can be performed in a single run-through (for the classical approach) or interrupted (for the SID approach). First, a nonmonitored patient presents with minor symptoms but remains clinically stable. The participant has to check the vital signs, ask the nurse for the history and the results of the physical examination, and order investigations. Second, the patient presents with acute collapse. The participant has to interpret investigation results while managing the acutely ill patient. Third, the participant must present a summary of the situation and a treatment plan to his supervisor while pursuing management of the patient. For the SID approach, the scenario is stopped at two times: (1) after ordering investigations, and (2) after receiving results of the investigations and prior to the call from the supervisor. The last stop occurs at the end of the scenario.

Based on these principles, the chosen scenario consists of an infant with disseminated herpes simplex virus infection presenting with secondary septic shock. A newborn will present with tachycardia and will evolve towards hypoxemia and hypotension, requiring intubation and volume expansion. Laboratory findings will reveal viral sepsis with leucopenia, thrombocytopenia, increased C reactive protein, and elevated liver enzymes. Finally, skin blisters will appear during the transfer of information from the participant to the supervisor and will confirm the diagnosis.

### Data Collection and Measurement Tools

#### Presimulation Questionnaire

This questionnaire will include demographic data (gender, age, year of graduation, number of previous experiences with simulation, learning style, and curriculum followed), degree of self-assessed subjective stress, and self-evaluation of clinical reasoning performances (both using a 10 point Likert-type scale question).

#### In-Simulation Clinical Reasoning Assessment

In constructing the simulation scenario and vignette, defined cues will be embedded to stimulate hypothesis generation using System-1 of clinical reasoning. After reading the initial patient presentation in the vignette, residents are asked to submit their diagnostic hypothesis by writing. Then, during the scenario, the participant’s verbalization (which is also stimulated by given cues from the nurse) of diagnosis coming to mind will be audio recorded. Both the written and audio-recorded hypotheses will be compared to the diagnoses generated by an expert panel that will be submitted to the same scenario. For example, the presence of thrombocytopenia during the data confirmation part should lead to verbalization of (1) bacterial infection, (2) viral infection, (3) intrauterine grown retardation, and (4) platelets immunization. The presence of skin lesions during the data confirmation part should lead to verbalization of (1) herpetic infection, (2) bacterial infection, and (3) varicella infection.

Iterative discussions supported by the Clinical Reasoning Assessment Tool during SID have been designed to allow development and exploration of System-2 of clinical reasoning regarding data collection, diagnostic hypotheses generation, new data interpretation, and management plan. In the classical approach, it is hypothesized that System-2 is discussed during the debriefing period after the simulation. Exploration of how both simulation approaches impact on performance of System-2 will be done during the semistructured interviews (see below).

#### Postsimulation Questionnaire

Postsimulation questionnaire (Multimedia Appendix 1) will assess residents’ self-reported improvement in clinical reasoning and level of satisfaction regarding the simulation approach (both using a 10 point Likert-type scale question). The questionnaire will be designed based on previous literature [63-65] and will be pilot tested with three residents. According to the simulation approach, residents will also complete 5 to 12 questions asking them to rate statements using a four-point Likert-type scale, ranging from 1 (strongly disagree) to 4 (strongly agree).

#### Semistructured Interviews

An individual semistructured interview will explore “how” and “why” students’ clinical reasoning ability develops through both simulation approaches (SID or classical). Interviews will be conducted using techniques inspired from the explicitation interview during which the interviewer supports the participant, without induction, toward the evocation of a specified experience [66]. After icebreakers, interviews will be constituted of two distinctive parts. First, the interviewer will explore the residents’ reasoning process during the simulation experience. Second, he will focus on residents’ perception of the simulation experience, including possible advantages and challenges of each simulation type, possible improvements to each methodology, and the perceived impact of SID on the participants’ learning. Interviews will be audio recorded and then transcribed for analysis.

### Data Analysis

This is an exploratory study that will aid in planning a future larger randomized controlled trial. The target population of residents enrolled in the General Pediatrics and Neonatal-Perinatal Medicine programs represents approximately 50 residents. Based on literature from qualitative research inquiry, the adequate sample size consists of the number of participants at which saturation of data is achieved. This well-described process permits cessation of recruitment when additional data does not bring new properties to unsaturated categories [67]. Different authors agree that saturation is reached after 20 to 25 participants [67,68].

Quantitative data will be analyzed using SPSS 20.0 (IBM SPSS, Chicago, IL). Data will be analyzed using descriptive statistics for all variables. For each Likert-scale type question, a significant cut-off will be pre-established. Comparison of positive versus negative responses will be done with the use of chi-square and Fischer’s exact test for nonparametric variables. Number, nature, and order of diagnostic hypotheses will be compared to the responses of a panel of 10 neonatologists from our hospital. A multivariate analysis will be used to investigate potential effects of graduation year, gender, and prior simulation experience on the residents’ evaluation of their clinical reasoning. Statistical significance will be defined as a probability value of <.05.

Qualitative data will help describe if SID allows better assessment of System-1 and 2 of clinical reasoning compared to the classical approach of simulation. Data from audio-recorded semistructured interviews will be analyzed using NVivo 9.0 software. Analysis will occur as data collection pursues. Recurring themes or distinctive aspects about each student’s response will be noted. These notes will be reviewed and expanded as the research continues until saturation of data. Data will be de-identified so that all participants will remain anonymous. Through content analysis, the information will then be categorized according to the principle of convergence [69]. The research team will use deductive analysis and review of all written transcripts.

## Results

This study is in its preliminary stages and the results are expected to be made available by April, 2016.

## Discussion

Clinical reasoning is an essential skill for everyday medical practice. However, many questions remain regarding how efficiency of the reasoning processes can be most accurately measured [16]. We believe that medical simulation could represent an effective environment for clinical reasoning assessment, as participants are immersed in an authentic and controlled setting [70]. Moreover, SID, an innovative approach to simulation, could provide a closer assessment of both System-1 and 2 of clinical reasoning by the combination of concurrent verbalization and iterative discussions at key steps of the reasoning process.

In this exploratory randomized study, comparing SID to the classical approach of simulation, we expect to find a higher progression of residents’ self-assessed clinical reasoning process with SID. More precisely, iterative discussions, by allowing reflection, will lead to improvement in System-2 clinical reasoning process while concurrent verbalization during management of the mannequin will enhance performance of System-1. Verbalization during the classical approach will also have a similar impact on System-1 clinical reasoning. Finally, residents will demonstrate a higher level of satisfaction in the SID approach of SBE.

This is the first randomized study comparing a new simulation approach to the classical mode for developing clinical reasoning skills. In addition, incorporating a qualitative piece to the study with the goal of exploring how each simulation approach impacts on residents’ clinical reasoning process is of great interest. Residents of different training levels are included in the study allowing to explore the phenomenon from perspectives of individuals with various levels of clinical reasoning performances. There are also a few limitations to the study. The small sample size does not allow for statistical generalizability of quantitative results. The absence of a robust pre and post assessment of residents’ clinical reasoning abilities might not portray the exact impact of each intervention. However, in the absence of a gold-standard tool in clinical reasoning evaluation, this exploratory study could provide detailed and useful preliminary data for the effectiveness of such a new simulation approach. Finally, the case specificity of one unique scenario could limit generalizability of the results.

The findings of the study will be of benefit to medical educators, training programs, and residents who participate in these programs. This study will further our understanding of the complexity of clinical reasoning, and how delivery of the curriculum should be modified to assist residents in better developing their clinical reasoning abilities in neonatology but also in others specialties. This study demonstrates the feasibility of a SBE session where scenarios are built according to clinical reasoning and reflective practice theories and help to put emphasis on clinical reasoning teaching and assessment. For medical residents, development of a simulation approach that assesses their clinical reasoning abilities will provide them with an opportunity to receive feedback about components of clinical reasoning which need to be improved. It will also provide medical teachers with an opportunity to better understand the students’ diagnostic process. Overall, insight into the clinical reasoning process by SBE may contribute to changes in medical education curriculum development and implementation. This may provide residents with better opportunities to develop clinical reasoning by SBE and become clinically competent doctors.

The future should concentrate on optimizing clinical reasoning assessment. A SID session could integrate a combination of well-described clinical reasoning evaluation tools such as script concordance tests [71] or clinical reasoning problems [72]. The validation of such an instrument could provide an essential educational tool for formative or summative assessment of medical students, whatever their level of training or their specialty. In a greater future, assessment of long-term retention of acquired clinical reasoning skills after exposition to SID should be explored once the basics have been settled.

## Multimedia Appendix 1

Post-simulation questionnaire.

## Abbreviations

NICU: neonatal intensive care unit
PGY: postgraduate year
SBE: simulation-based education
SID: simulation with iterative discussions
