# Supplementary figures and images for: Exploring a New Simulation Approach to Improve Clinical Reasoning Teaching and Assessment: Randomized Trial Protocol
Source: JMIR Res Protoc. 2016 Feb 17;5(1):e26. doi: 10.2196/resprot.4938 (PMC4776024; doi:10.2196/resprot.4938)

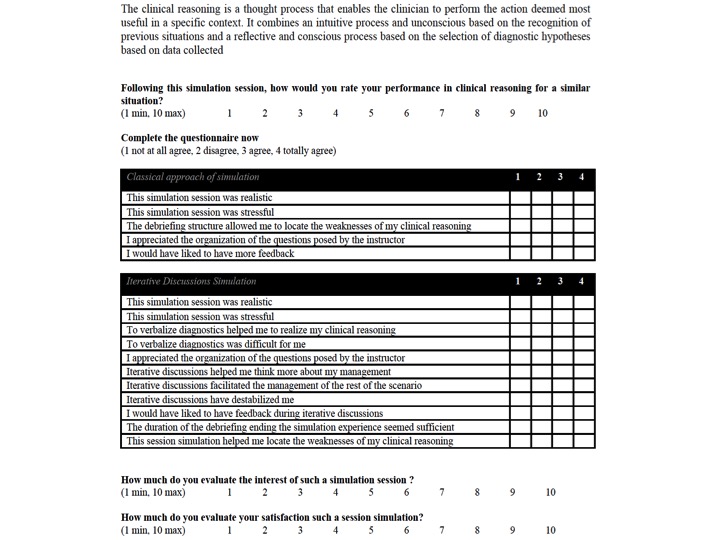

Supplement: Supplementary file 1 [file resprot_v5i1e26_app1.jpg]
